# Changing perspectives on corporal punishment in schools: Insights from Ugandan young people

**DOI:** 10.1177/09075682251392494

**Published:** 2025-10-31

**Authors:** Jenny Parkes, Daniel Carter, Rehema Nagawa, Junior Brian Musenze, Joan Ritar Kasidi, Simone Datzberger, Tvisha Nevatia, Janet Nakuti, Amiya Bhatia, Dipak Naker, Karen Devries

**Affiliations:** 1 4919 Institute of Education, University College London, London, UK; 2 228093 Department of Population Health, London School of Hygiene and Tropical Medicine, London, UK; 3 47968 Medical Research Council/Uganda Virus Research Institute and LSHTM Uganda Research Unit; 4 735464 Raising Voices, Kampala, Uganda

**Keywords:** corporal punishment, violence, discipline, Uganda, Africa, schools

## Abstract

This paper contributes to decolonising ways in which corporal punishment has been understood and represented in Africa, through engaging with the complex views of young Ugandans about corporal punishment in schools. Conceptualising time as non-linear, and violence as a web of power, our analysis of longitudinal, mixed method data traces how physical punishment functioned to maintain the generational order, and as a regulatory practice in schools. While young people adopted critical standpoints against corporal punishment, structural conditions and violence in schools evoked feelings of insecurity, that could generate resistance to change. Our analysis problematises assumptions that legal bans alone will create meaningful change.

## Introduction

Since the late 20^th^ century there has been concerted global and national mobilisation against corporal punishment. Bans have been promoted by international human rights legislation, with the UN Convention on the Rights of the Child (UNCRC) (1989) requiring states to ensure that no child is subject to ‘degrading treatment or punishment’, including from 2006 ‘any punishment in which physical force is used and intended to cause some degree of pain or discomfort, however light’. The African Charter on the Rights and Welfare of the Child (1990) required that ‘domestic discipline is applied with humanity and in a manner consistent with the inherent dignity of the child’ (Article 20 (1) (c)), with its Committee in 2018 supporting prohibition of corporal punishment in all settings. Ending all forms of violence against children is a target (16.2) of the 2030 Agenda for Sustainable Development. As well as violating children’s rights and dignity, corporal punishment has been associated with physical, academic, mental health and behavioural problems for children (Lansford et al., 2002; WHO, 2025). It has been prohibited by law in schools in 136 states, and in all settings in 65 states^1^.

However, there is limited evidence to suggest legal bans on corporal punishment are effective and the practice remains widespread in schools in many contexts (Gershoff, 2017), including in Uganda (Devries et al., 2015; Ministry of Gender, Labour and Social Development, 2018). A global review found no clear pattern of lower levels of corporal punishment in countries where bans were in effect, and that corporal punishment did not appear to be reducing over time (Heekes et al., 2022). Where corporal punishment has reduced, as in Sweden, there is evidence that this is because of public attitude shifts that pre-dated legal changes, and not because of the bans themselves (Roberts, 2000; WHO, 2025).

In Uganda, Article 24 of the Constitution (1995) gave children the right to be educated without humiliating and degrading treatment. Corporal punishment was discouraged in schools by Ministerial Circular No. 15/2006, and prohibited in 2016, when the Children Act was amended, with a person who commits corporal punishment “liable to imprisonment for a term not exceeding 3 years or to a fine not exceeding one hundred currency points or both” (article 106A). In 2016, Uganda became a pathfinder country with the Global Partnership to End Violence Against Children, committing the Government to accelerated action towards SDG target 16.2, but corporal punishment is still lawful in homes. The Government has produced guidance for schools, including the National Strategic Plan on Violence against Children in Schools (NSP VACiS) (2015-20) and Reporting, Tracking, Referral and Response (RTRR) Guidelines, with schools expected to have School Disciplinary Committees, a Senior Man/Woman Teacher coordinating VACiS, and child-friendly reporting such as suggestion boxes. Recent analyses by the Safe to Learn Coalition (2020, updated in 2023) have praised the legal and policy framework, but noted that VACiS work in education is largely donor funded, enforcement of the law is weak, and implementation of the guidance is uneven^2^. Implementation efforts have been mobilised by civil society organisations working with local authorities. Studies of these interventions have problematised simple linear routes between legal change and punishment practices, and highlighted the importance when working with schools, of deep engagements over time with institutional cultures, teacher-pupil relationships and school structures (Kyegombe et al., 2017), and engaging parents (Lokot et al., 2020).

Implicit in many interventions on corporal punishment is an uncritical framing of lack of ‘progress’ as a deviation from a civilising continuum (Balagopalan, 2024), with children, caregivers and teachers in Africa positioned as falling behind on a linear road to safe schools, as we discuss further in the next section. Challenging this view, our goal in this paper is to delineate how, in the context of Uganda, variegated, complex factors inform and shape positions of young people who are in or have recently navigated the school systems, amidst legal changes. Drawing on data from a longitudinal, mixed method study, we are interested in learning from young people, and their caregivers and teachers, about why corporal punishment in schools persists, or changes, over time. Through this analysis, which conceptualises violence as a web of power and time as non-linear, the paper contributes to scholarship that decolonises the way corporal punishment has been understood and represented in Africa.

## Analyses of corporal punishment in Africa

Our analysis builds on a rich body of scholarship, mainly from Africa, that brings decolonial perspectives to investigate the persistence of corporal punishment in schools and homes. One strand of this work has argued that corporal punishment should be viewed not as an indicator of backwardness of African cultures, but as escalated by colonial practices and values. Assie-Lumumba (2012) traced how, for missionaries and colonizers, education became a tool to ‘conquer’ the African mind, with classrooms sites of colonial domination, subordination and training in punctuality and obedience. Muhangi (2017) argues that, though corporal punishment was practised in pre-colonial Uganda, its embedding within the education system can be attributed to British colonialism. In the context of colonial Kenya, Ocobock (2017) traces how an age-based disciplinary regime operated to impose generational authority by elders, including through corporal punishment, to control youth, particularly young men, and persisted through the uncertainties of the late colonial period and the post-colonial state’s project of decolonising and nation-building.

Contemporary movements against corporal punishment have also been critiqued for their imperialist imposition of Western provincial norms about children’s rights and discipline. Pupavac (2011) charts how historically social conditions in post-industrial societies influenced ‘softening’ of discipline norms, which international child rights advocacy then tried to impose globally. A cross-cultural analysis found corporal punishment to be more commonplace in societies with higher levels of poverty and inequality, colonial histories, and undemocratic political decision-making, concluding that physical punishment might be practised to prepare children for living in a society with imposed inequality (Ember and Ember, 2005). In a study of Maasai communities in Kenya, Archambault (2009) argued that physical punishments played a complex role in identity development, marking a boundary between childhood and adulthood and preparing children for harsh material conditions and pains of adult life. In Zanzibar, Fay (2019) critiqued how child rights governance operated as a hegemonic system governed and regulated by international organisations, enacted by states and NGOs, neglecting contextualised ideas of childhood, and frequently resisted by child protection actors.

Critical of deficit depictions of African childhoods, Twum-Danso Imoh (2013) found that views on punishment among children and caregivers in Ghana drew on collective value systems of reciprocity, respect and responsibility, with corporal punishment viewed as a means to instill values and signify care, though they were critical of excessive or unwarranted punishment. Caregivers in a Tanzanian study viewed some beating as protective in an unsafe neighbourhood, but excessive beating as abusive (Frankenberg et al., 2010). Reviewing evidence on social norms and punishment in lower and middle income countries, Lokot et al. (2020) concluded that while in most studies, normative beliefs supported and sustained corporal punishment, there were also norms that set limits on its severity.

School-based studies have explored why teachers continue to practise corporal punishment despite bans. Drawing on Foucault’s ideas about governmentality to investigate teachers’ responses to the South African government’s ban on corporal punishment, Govender and Sookrajh (2014) found that authoritarian practices persisted, with power and control exerted through physical and non-physical techniques, like surveillance and testing. In another South African study, learners viewed corporal punishment as necessary to maintain discipline, peace and academic achievement, while teachers perceived the ban as a symbolic attack that was then turned on the learners in the form of humiliating punishments (Payet and Franchi, 2008). Studies have explored how corporal punishment reproduces hierarchical relationships, shaping relationships between students and teachers (Rojas Arangoitia, 2011), including gender relations (Turner et al., 2024). Stress and frustration among teachers in contexts of material hardship and poor working conditions, has been associated with persistence of corporal punishment in studies in South Africa (Govender and Sookrajh, 2014; Hunter and Morrell, 2021), and Tanzania (Hecker et al., 2018), and in Kenya its use by teachers has been associated with high pressure exams (Vanner, 2019).

This literature provides insights into why efforts to reduce corporal punishment, including legal bans, may have very limited impacts. They invite us to question whether the expectation to end corporal punishment in linear ways – through the imposition of a ban or an externally implemented programme - is itself colonial, reproducing hierarchies of power that dismiss complex structural conditions and social relations in schools, and ‘non-western’ constructions about childhood, interdependency, reciprocity, and protection (Twum-Danso Imoh, 2022). In general, the literature has been stronger in showing what deters than what facilitates change, and few studies have tracked perspectives over time.

## Violence as a web of power through time and space

Analyses of corporal punishment tend to refer to caning or beating, slapping, shaking, kicking or pushing, and often include psychological forms of punishment, including humiliation, ridicule, or withdrawing love, which tend to coincide with physical forms (Lokot et al., 2020). Our conceptualisation is multi-dimensional (Parkes et al., 2013; Paulson and Tikly, 2023; Scheper-Hughes and Bourgois, 2004), and builds on feminist and postcolonial scholarship that links violence, power and subjectivity. We understand subjectivity as ‘the felt interior experience of the person that includes his or her positions in a field of relational power’ (Das and Kleinman, 2000 p.1), and we conceptualise violence metaphorically as a shifting web of power. The outer threads of the web denote structural violence of inequitable and oppressive systems and institutions, such as socio-economic, political and education structures, that underpin harsh punishment at the heart of the web. They connect to the inner threads – the everyday interactions through which relational power shapes subjectivities, and through which structures of oppression may be internalised. They depict the performative ways in which punishment practices operate as a societal mechanism of control and regulation, intended to deter through fear or humiliation, and symbolic violence (Frevert, 2020).Through repeated practice, assumptions about physical pain and humiliation as a means for adults to control children become inscribed on young bodies and minds, coming to be perceived as natural, part and parcel of the order of things. Violence may be used to marginalise and subjugate, generating insecure subject positions for those whose subjectivities are excluded or denigrated (Butler, 2004; Fanon, 1967; Spivak, 1988). Viewed through a multi-dimensional violence lens, the studies discussed above show how corporal punishment practices are bound up with histories, continuing colonial legacies, contemporary global forces and subjugating networks of power that seep into subjectivities and relationships within classrooms.

But while the web ensnares, it is constantly in motion, with power dynamics shifting across time and space. Young people may occupy multiple, and sometimes conflicting, subject positions as they move between different social spaces, for example, of the family, school or workplace. Our longitudinal analysis views Time not just as chronological, but as culturally constructed, subjective and performative (Butler, 2004; Lapping, 2020). As they reflect on their experiences and perspectives about punishment, young people may speak from subject positions layered with time as historical, maturational, memories of the past, the continuing present, and imaginings of a future yet to come. Within this framing, we conceive change, not as linear, but as emerging in complex, contradictory ways (Saldana, 2003), as young people navigate positionings through time and space.

## CoVAC study

The analysis for this paper stems from the Contexts of Violence in Adolescence Cohort Study (CoVAC) (2018-2023).^3^ CoVAC is a mixed method longitudinal cohort study that aims to build understanding on how family, peer, school and community contexts affect young people’s experiences of violence in adolescence and early adulthood. The study was located in Luwero, a district of central Uganda with a multi-dimensional child poverty rate of 41% (Ugandan Bureau of Statistics 2024). Bordering Kampala, it spans rural and urban areas, with the main source of income coming from subsistence farming. In this context, the generational shift between childhood and adulthood is marked less by age than by transitions from schooling, home or into marriage, and the boundary between childhood and adulthood is blurred by many school-going young people also engaging in paid and unpaid labour (Abebe and Ofusu-Kusi, 2016). We therefore use the term ‘young people’ when describing our participants, in recognition of the fluidity and variability of their generational positioning. Young people from 42 primary schools were invited to participate in 2014 following a school-wide violence prevention intervention. More information about CoVAC’s study design is available elsewhere (Devries et al., 2020). Briefly, quantitative data were collected at three time points (2014, 2018, 2022). For this paper, we analysed data from 3431 young people (aged 11-14), who participated in the 2014 face-to-face survey, and 2773 of these young people (aged 15-18) surveyed in 2018.

Qualitative data were collected for 2-3 months each year from 2018 to 2022, with 36 core participants (18 female, 18 male), aged 15-17 (in 2018), and, guided by the core participants, with their teachers, caregivers, peers, and other stakeholders. Each young person engaged in a series of biographical narrative interviews, focus groups and unstructured discussions with their ‘key’ researcher, who was Ugandan and usually same sex (always for females), and with whom they built strong research relationships through multiple encounters. Data were translated (from Luganda to English), transcribed, with pseudonyms drawn from the context, coded thematically using NVivo, and with biographical narratives co-constructed over time for each participant. The study followed CoVAC’s ethics protocol, with agreed procedures for managing abuse disclosures and support from a local counselling organisation^4^.

To analyse experiences and perspectives on corporal punishment, we developed a layered mixed methods analysis. Quantitatively, using R v4.3, we compared survey data from 2014 to 2018 on experiences of and attitudes towards physical violence by teachers among school-going young people. We explored whether the prevalence of physical violence by school staff had changed, inequalities in prevalence, and whether there were changes over time in attitudes supporting corporal punishment.

The qualitative analysis explored the structural dynamics and discourses attached to punishment practices, how these varied between participants and over time. While the analysis drew across the rounds of data collection, we focused particularly on data collected in 2022, when young people were invited to reflect on emerging findings, including the range of viewpoints supporting or disagreeing with the ban. As the analysis progressed, we were struck by the variability not just between young people, but often within a single interview. To understand the complexity of their views, applying the multi-dimensional framing of violence, our analysis delved into their structural and subjective positionings within the web of power.

## Quantitative data: Persisting practices and shifting perspectives?

Between the 2014 and 2018 surveys, there were significant changes in young people’s lives, as they grew older, transitioned from primary to secondary or out of school, and with corporal punishment in schools finally banned by law in 2016. By 2018, 1248 of those surveyed were no longer in school. Survey responses of those still in school in 2018 showed that corporal punishment remained commonplace in schools, but young people’s attitudes towards such punishments shifted markedly (Table 1).

**Table 1.** Prevalence of physical violence from a teacher (past year) experienced by school-going young people.

Past-year physical violence from a teacher^a^	2014 (n = 3431)	2018 (n = 1525))
No	802 (23.4%)	314 (20.6%)
Yes	2629 (76.6%)	1211 (79.4%)

^a^In 2014 and 2018, measures of physical violence include being slapped with a hand, having an ear twisted, hit with an object (incl. caning), doing forced labour, being made to stand or kneel, and severely beaten up. In 2014 only, also included were being hurt or caused pain, having an arm twisted, hair pulled, fingers hit or crushed, head knocked, hit with a fist, having an object thrown at, kicked, being forced outside, being burnt, having food taken away, forced into doing something dangerous, choked, tied up with a rope or belt, and cut with an object.

With over three-quarters of school-going young people having experienced physical violence from a teacher in the past year in 2014 and 2018, these data appear to indicate little change in school punishment practices. But in 2018, those still in education had moved to secondary schools, so any changes in primary school practices would not be evident in our data. Nevertheless, it is clear that most young people continued to experience corporal punishment as they moved through different phases of schooling. As shown in Table 2, such punishments were unequally experienced.

**Table 2.** Prevalence of past year physical violence by teacher by school-going young people’s characteristics.

	Past year physical teacher violence 2014		OR (95% CI)	Past year physical teacher violence 2018		OR (95% CI)^a^
	No violence	Violence		No violence	Violence	
Girls	429 (23.2%)	1424 (76.8%)	REF	193 (22.9%)	651 (77.0%)	REF
Boys	373 (23.6%)	1205 (76.4%)	0.97 (0.83, 1.14)	121 (17.8%)	560 (82.2%)	1.37 (1.06, 1.77)
No functional difficulty/disability	642 (23.8%)	2052 (76.2%)	REF	240 (21.6%)	869 (78.4%)	REF
Functional difficulty/disability	160 (21.7%)	577 (78.3%)	1.13 (0.93, 1.37)	74 (17.8%)	342 (82.2%)	1.28 (0.96, 1.70)
3 or more meals in last 24 h	392 (25.8%)	1126 (74.2%)	REF	169 (21.4%)	619 (78.6%)	REF
Less than 3 meals in last 24 h	408 (21.4%)	1501 (78.6%)	1.28 (1.09, 1.50)	144 (19.6%)	592 (80.4%)	1.12 (0.87, 1.44)

^a^OR = Odds Ratio, which is the odds of experiencing past year teacher physical violence in one group compared to the odds of experiencing past year teacher physical violence in the reference group, unadjusted for any confounding variables.

**Figure 1.**
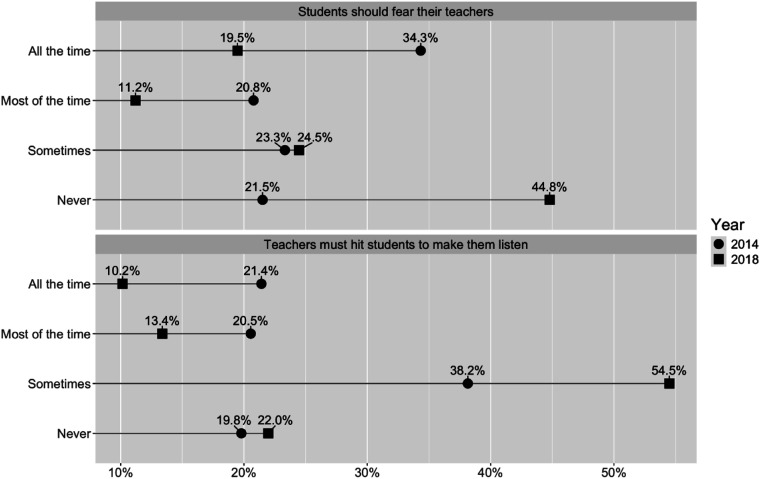
Changes in school-going young people’s attitudes between 2014 and 2018.

Girls and boys faced similarly high levels of physical punishment by teachers in 2014 (average age 13). In 2018 (average age 17), there was little change for girls, but boys faced higher levels than in 2014. Though small sample sizes - in some part due to the high prevalence of physical punishment - limit the conclusiveness of these findings, we observed that at both time points, levels of physical punishment were higher in young people reporting functional difficulty or disability and higher in young people who had eaten fewer than three meals in the past 24 hours. In contrast to the prevalence data, the data on young people’s attitudes to school punishments indicated marked changes between 2014 and 2018 (Figure 1), with school-going young people becoming over time significantly more critical of such punishments.

Between 2014 and 2018, there was a marked shift away from agreeing that ‘students should fear their teachers’, and ‘teachers must hit students to make them listen’ all the time/most of the time. In 2018, while over half the young people felt that corporal punishment was sometimes needed, they appeared to have developed tighter boundaries on what they deemed acceptable punishments, and were much more critical of fear as a pedagogical tool. To understand these shifting patterns in young people’s attitudes further, we turn to our qualitative data.

## Corporal punishment and the generational order

Echoing these quantitative findings, across the rounds of qualitative data collection, young people expressed varying views about punishment practices. Often they supported physical punishment as part of the disciplinary repertoire in home and school, so long as it was deemed fair, moderate and proportional. Many felt that corporal punishment had reduced over time, but some were critical of the changes, as they viewed these disciplinary practices as an inevitable component of raising children that maintained the generational order. Within the reciprocal relationship between adults and children, young people were expected to learn and perform humility, obedience and respect, and caregivers, including teachers acting *in loco parentis*, to discipline in order to uphold these values and maintain order. Such views were expressed often by young people who were out of school. Kiprotich, for example, reflected on why he had become increasingly supportive of caning:

Kiprotich (age 22): As a child you wouldn’t want to be caned.

Joan (Researcher): So do you think your opinion on it has changed?

Kiprotich: Yes

Joan: What do you think has brought about that change?

Kiprotich: From what I have seen and what I am, caning is good.

Joan: What makes it good?

Kiprotich: Because when I was still young, I stole my grandfather’s money and he caned me. Young me at the time thought caning was a bad thing, but since now I am not a thief, I think it is a very good thing.

For Kiprotich, the transition to adulthood, marked by having to drop out of school due to economic hardship, provides temporal distance from the pain of punishment, that had evoked criticism by ‘young me’, and he resignifies caning as a pedagogical tool. His words ‘from what I have seen and what I am’ neatly express the layers of time – his memories of the past and the continuing present - that have influenced his views. Kiprotich also has childcare responsibilities for his younger siblings, and told Joan how he disciplines with up to three canes or by giving chores, like fetching water. Archambault (2009), reflecting on pain in punishment in Maasai schools in Kenya, noted that it may be in young people’s interests to support these practices, as they too will be able to exercise power through pain as they get older. For Kiprotich, exercising punishment as his elders did demonstrates that he is performing responsible adulthood.

Sam (age 20), who worked in subsistence farming since dropping out after primary schooling, was also critical of perceived reductions in caning in recent years: ‘*but you can’t just let the child grow wild without beating him sometimes*'. Like Kiprotich, he viewed childhood beatings as contributing to his present identity:

Brian (Researcher): Why do you think some children are for beating and the rest are against it?

Sam: That would require now to think about it in the way that each thinks is right for them. Because for us who were beaten are far different from those children who were not beaten. You could even see from our dressing. Someone can dress inappropriately because they were not beaten when young. Those who are not beaten are the troublemakers. I think a child must be beaten.

Struggling economically throughout his life, Sam positions himself in the continuing present as a responsible, respectful young man, whose identity has been shaped through memories of childhood beatings. His support for beating as integral to child socialisation may be sharpened by his lived experience of being denied the tropes of ‘modern’ life, symbolised here by fashionable clothes and absence of disciplinary control.

Several young people and caregivers attributed what they perceived as increased unruliness among children to changes in laws and policies on children’s rights. Kalumba’s father, Noir, reflected on changes since his childhood, when ‘*teachers would beat you like crazy*’:

Noir: I think this has changed for the children of these days, but again the way it has changed is not for the good, there are many laws, there is no balance of the laws. The children are over privileged against the parent and the teacher. To the extent that a child can contend with the teacher in class. Sometimes the child can even out of anger leave class because he has had a disagreement with the teacher. I think that the laws are so many and are bent to the children’s side. There should be a balance.

While critical of harsh punishments recollected in his early life, Noir also criticised recent government policies that he viewed as disrupting the generational balance of power so that teachers’ (and parents’) authority is undermined. Kayondo’s mother too lamented the effects of the ban on children:

There is a child a few months ago who told me that if his father was to dare to touch him, he would go to police because the state said they were not to be beaten. You could see that the child has this unrealistic ego that pushes him to do things because he has backup from the state.

For these caregivers, the imposition of laws and policies emphasising children’s rights, and illegitimising physical punishments, was perceived as aggravating discord between increasingly unruly, argumentative children and their parents and teachers, no longer able to exercise their responsibilities.

Some caregivers, however, supported the changes. Chekurut’s father was glad that beatings had reduced, due to growing public awareness of its ineffectiveness to change children’s behaviour and to legislation. Kalungi’s mother reflected on shifts in her own views and practices, as the stress of material hardships in her life eased, and with support from others in the neighbourhood:

When they were small, I used to cane them, for now I talk to them. I speak to them and I advise them. I tell them that I don’t want that, or I want this to be done. I also tell them that if they go on doing something that I don’t want, I won’t be happy. I even threaten them that if they don’t listen, I shall take them to the police and the police will work on them for me. I can take them to the police and I ask them to advise my children because if it gets too hard for me and I cannot advise them, I just do that. There is a woman at the police who always talks to them for me, she tells them that they have to listen to their mother. I also call pastors to them and they advise them for me.

These caregivers questioned the effectiveness of corporal punishment to maintain the generational order, and proposed the logics of discipline and punishment being held in place in other ways – through talking, or verbal interventions by authority figures in the community. For them, the legal ban supported, rather than undermined their caregiving, and they perceived it as helping to reduce excessive punishments in schools.

## ‘Regulated beating’ in schools

As a routine disciplinary practice in schools, corporal punishment is intended to work performatively (Rojas Arangoitia 2011). Often performed publicly, in front of class or school, its repeated practice, experienced and witnessed, shapes social relations, defining students as lacking authority and voice, penalising and stigmatising those who transgress. While young people were critical of excessive or unfair punishments, often they took for granted and accepted its regulatory function, as Anna (age 19) explained, using the example of students bringing illegal drugs to school:

These two would eat and smoke drugs. If you stop beating, how do you expect such an act to stop? We do come from different families, that means if someone has such a character, they can pass it on to the other students who are good. You can’t control all students, some need to be beaten. If for example, the senior 4s do an exam and fail, and then they fail again and you do nothing, that means they shall take it for granted and continue to fail. I think that there should be some regulated beating to help these children.

For Anna, bringing drugs to school or failing exams – characteristics she associated with families different from her own – justified physical punishments.

Also critical of the ban was a teacher in Nkola’s school, who viewed corporal punishment as necessary to maintain discipline in overcrowded classrooms, in the context of the policy of universal secondary education (USE):

No caning is okay for a school which has very strong policies on discipline like church founded schools but in a public school where you have all religions, everyone worships however they wish, where there are no strong roots of religion like church founded, a USE school where you are gathering children from all backgrounds and behaviours, sometimes it is very hard for you to curb that indiscipline with the growing numbers in the school. Because when you receive very many students, the problems come on how to maintain that number. So when you totally put aside the stick, controlling the discipline becomes very hard, sometimes you just need to know how to regulate it.

Poor working conditions in underresourced public schools made it difficult for teachers to maintain authority, and to change their disciplinary practices by ‘putting aside the stick’. It should be noted though, that, in young people’s accounts, corporal punishment was also practised in some private and faith schools, which were sometimes preferred by parents because of their use of physical discipline.

The varying practices between schools, and high mobility between schools, meant that young people were often exposed to multiple institutional approaches, with the potential to disrupt their perspectives. Kalungi (aged 20) reflected on how his own views had changed:

Brian: Do you think your perception about punishment has changed a lot?

Kalungi: It has changed. Growing up and going to new levels, has caused me to think differently about punishment. While in primary school, beating was almost normal, this is not the case with secondary school. Those days were corporal punishments, you could kneel down in the front of class and carry bricks on your hands. I think this has changed.

[….]

Brian: What do you think of children saying that beating with one or two canes is fine enough?

Kalungi: I don’t think that can change anything. Beating does not have any impact; it can cause pain but it cannot change someone’s character. I think that instead of beating a child, you could instead talk to them so they can change their character or mentality.

In his memories of past punishments, Kalungi recognises the performative function of corporal punishment shaping views when at primary school. His current school, in contrast, was a high achieving secondary school with a strict system of fines and suspensions for rule breaking but rarely using corporal punishment. He perceived his own views as having changed with maturation and lived experiences in different schools, so that he now views corporal punishment as ineffective.

Kalungi’s evolving perspectives were also shaped by his relationship with his mother, who, as we discussed above, had become increasingly critical of physical discipline herself. Kalungi also recalled the Good Schools Toolkit intervention implemented several years ago in his school by the NGO, Raising Voices:

It did a lot for me, I did not even know about the good schools, you could think that a good school is one with nice buildings, expensive things, and brilliant teachers, but instead, a good school is one that has a good studying environment, where all students are taken equally, no segregation among students and teachers must interact with students so they can find ways of helping children lead good lives. Helping them academically other than beating them.

Kalungi’s reflections articulate the significance of the broader school ethos in shaping perspectives. From a relatively secure subject position as a successful, high achieving student, he was able to reflect critically on the potential for more equitable relations in schools to support learning.

Like Kalungi, Apio (aged 20) was a high achieving pupil, who had managed to stay in secondary school through sponsorship, and was critical of corporal punishment. She narrated how she had managed to avoid being punished when she was unwell through negotiating with school staff:

I missed an extension, I tried to explain to the matron who allowed me to go and rest, I had severe cramps. But this teacher came and wanted to beat me for missing an extension, I tried to explain but they could not understand. I asked him to allow me to go and explain myself to the administration, which he did and I dodged the beating.

For Apio and Kalungi, their relatively secure present-day positions as learners within schools which operated disciplinary systems that at least awarded them some voice, may have contributed to their criticality. Others, however, occupied much less safe positions, to which we turn next.

## Insecure subject positioning within school contexts

When schools reopened after 2 years following the COVID pandemic, several young people rejoined different schools. While this in itself could be unsettling, erratic disciplinary practices seemed to aggravate insecure subject positionings, generating ambivalent views about corporal punishment. Nkola recalled primary school experiences of beatings and kneeling for failing tests, but when she moved to secondary school, caning was rare. After the pandemic, no longer able to cover the costs of her previous boarding school, she (age 17) moved to a local government school. Here she was bullied and humiliated by male students, and beaten repeatedly by staff for lateness, after leaving home each day at 5 am to walk the long journey to school. She was critical of teachers humiliating and discriminating against students: ‘*Then there is one who gives you a punishment, and still gets back to class to speak about it among the students, verbally insulting you, uses slandering words, talking about the student’s background status as they are poor, and speak against that student*'. She was not aware of any school disciplinary committee, nor of senior teachers to whom she could report.

Though deeply critical of the school’s disciplinary practices, she did not, however, support banning corporal punishments:

I say they should remain in school but make them a bit light because if they are there right now but still the students break the rules, then madam, imagine if they are removed completely what will happen, so they should be there but make them light.

Nkola’s views are influenced by her shifting positioning as a learner within institutions that have deployed varying punishment practices. Though she much preferred her former secondary school, where an effective disciplinary system banned the practice, the violence in her present school – by peers and teachers – rendered corporal punishment as simultaneously threatening and protective. Perhaps aware of the ambiguity, she resolves it by advocating ‘making them light’. Her views are shaped by memories of the past, overlain by a continuing present in which erratic, unpredictable use of violent punishments threatens both her physical safety and her former subject positioning as an effective learner.

In some other cases, young people complained that what appeared to be compliance with the law, in banning corporal punishment, was a smokescreen by staff for neglect and abrogation of their responsibility for young people’s safety. Anna, for example, was concerned that, although corporal punishment had reduced with the arrival of a new headteacher, the previously punitive discipline system was replaced by a ‘laissez-faire’ ethos, that permitted sexual activity, including between male teachers and schoolgirls, and left pupils like Anna feeling vulnerable and unsafe.

Insecure subject positioning was also evident in teachers’ narratives, as expressed by a teacher in Nakafeero’s school:

You have to get hold of the stick, because they say that spare the rod and spoil the child, you may look and see that school students who are not caned they are spoilt morally. For them they know that they are not supposed to be beaten, that if a teacher does beat her she will go and report her, which will result in the child not respecting you and still they will not listen to whatever you will tell her. But I want you to go and follow the schools where students are being caned, you will notice that their discipline will be very good.

For this teacher, banning corporal punishment was viewed as disturbing the balance of power in ways that increase the vulnerability of teachers, both to unruly pupil behaviour and to the prospect of being reported and sanctioned if they do use corporal punishment. She perceived the detrimental effects of children’s exposure to ‘global’ influences, including adopting negative behaviours from the internet, and policies against caning, as undermining teachers’ authority: ‘*In this era of technology, these children have access to phones, they have internet and they see what happens globally so they engage in a lot of things so sometimes you need some bit of force'.* For these teachers, insecure in their own positioning, the ban could be perceived as undermining their subject positions as effective teachers, who may then deflect the symbolic violence onto pupils through harsh and humiliating punishments.

## Conclusion

Through a novel longitudinal, mixed methods analysis, this paper adds to critiques of the positioning of African children, caregivers and teachers as falling behind on a linear path to violence prevention (Twum-Danso Imoh, 2022). Policies, including the legal ban in 2016, do not seem to have led to marked reductions in its practice in schools in this context, with most young people continuing to experience corporal punishment as they moved through different phases of schooling. But the young people participating in this study in Uganda conveyed complex, multi-faceted and shifting viewpoints, as they reflected on experiences, policies and practices of corporal punishment through their lives.

Theorising violence as a web of power and viewing corporal punishment through a non-linear time lens enabled us to analyse layers of power that hold corporal punishment in place. Corporal punishment was often viewed as key to maintaining the generational order, part and parcel of adult-child relations in which children were expected to learn and perform humility, obedience and respect, and adults, including teachers acting *in loco parentis*, to discipline to ensure these values are upheld and order maintained. Economic hardship was a barrier to change. Young people who were out of school, usually because of the costs of schooling, recalled disliking harsh punishments in their past school lives, but challenges in their ongoing present lives could make it hard for them to imagine beyond these perspectives of punishment as performing responsible adulthood. For young people still in school, repeated practices of corporal punishment shaped social relations, and those who transgressed could be penalised and stigmatised. Through this performative inscription on bodies and minds, some young people took for granted and viewed as legitimate its regulatory function. But practices in schools varied, and young people’s views were shaped in complex ways through memories of the past, alongside and overlain by the continuing present of their lived experiences in schools.

A critical finding is how insecure subject positioning within the web of power could generate resistance to efforts to stop corporal punishment. Underresourced, overcrowded schools and high-stakes exam systems, high pupil mobility due to economic insecurities in the COVID pandemic, and weak school discipline systems generated stressful working conditions for teachers and students. Young people who felt insecure and unsafe in schools with harsh, unpredictable discipline, and where they encountered peer violence, could view corporal punishment as both threatening and protective, and be fearful that banning it could increase their vulnerability. For teachers, struggling to fulfil their professional duties and maintain order in these conditions, government policies on children’s rights, and in particular corporal punishment bans, could be seen as undermining their capacity to teach or care. Global media, technology and government policies were viewed by some young people and adults as producing increasingly unruly children, alongside punitive threats to caregivers or teachers resorting to ‘tried and tested’ punishments. In this way, the imposition of the ban could be seen as a symbolic attack on teachers or parents (Payet and Franchi, 2008), that could in turn generate harsher punishments or the abrogation of responsibility for discipline or care. Thus, the web of violence entailed structural and symbolic relations that constrained the capabilities of young people, their teachers and caregivers, and reinforced corporal punishment practices.

But in 2018, school-going young people were markedly more critical than they had been in 2014 about the use of physical punishments and fear as pedagogical practice in schools. For some, their experiences within different institutional contexts enabled them to reflect on how their own viewpoints had changed to adopt more critical standpoints against corporal punishment, endorsing the ban, and advocating for dialogue and negotiation in adult-child relations. Such views were expressed most by young people who felt secure in their present positioning as senior students within a school with what they perceived as an effective disciplinary system – where there were non-violent alternative disciplinary approaches in place, and an institutional culture that fostered student voice and a sense of belonging. When they were embedded in a system supporting their views, including within their family and community relationships, they could feel safer to challenge, or unravel threads within the web of power that hold corporal punishment in place.

There are important implications for policy and practice. Our findings indicate that externally imposed, punitive interventions, such as corporal punishment bans, will not succeed in eradicating its practice without also tackling the surrounding webs of power. Violence stifles and closes down opportunities for dialogue and debate (Paulson and Tikly, 2023). Interventions need to identify the sources of insecure subject positions, and to work at multiple levels to address underlying material, structural and institutional forces, and to create conditions for more secure subject positions, conducive to questioning and deliberating in this contested space. Economic resources are needed for schools and families to support the aspirations of teachers and caregivers to foster learning, care and respectability. Schools need support to develop institutional cultures with alternatives to corporal punishment for them to be able to enact the ban in a sustainable manner. Teachers need to be supported to use positive discipline techniques that are non-violent and can still help in creating order, enabling them and their students to feel secure, and able to build stronger teacher-student and student-student relationships. Interventions need to extend beyond the school grounds, to engage caregivers and young people out of school, as they become parents themselves. This approach requires not only enforcing bans but also preventing and addressing violence through a more integrated and comprehensive strategy that includes education, community engagement and multiple dialogues on how to promote safe, inclusive learning environments for young people.

## Data Availability

Anonymised data will be offered to the UK Data Service, and made available through our university research data repositories. The research team will have exclusive use for 10 years after data collection.

## Notes

1. Ending corporal punishment in schools to transform education for all children https://endcorporalpunishment.org/wp-content/uploads/2023/05/Ending-corporal-punishment-in-schools-to-transform-education-for-all-children.pdf (retrieved 21/11/2024).

2. Safe to learn progress 2019-2023 Uganda https://www.safetolearncoalition.org/media/1736/file/Uganda_-_Safe_to_Learn_Progress_2019-2023.pdf; https://www.safetolearncoalition.org/media/351/file/STL-Uganda-Diagnostic-Exercise-2020.pdf. Retrieved 15/4/2025.

3. CoVAC is a research collaboration between London School of Hygiene and Tropical Medicine (LSHTM), UCL IoE (Institute of Education), Raising Voices, and the MRC/UVRI & LSHTM Uganda Research Unit, funded by the UK Medical Research Council.

4. The project has ethical clearance from UCL Institute of Education (REC 1091, Data protection registration number: Z6364106/2018/05/144 social research), the LSHTM, Uganda Virus Research Institute (UVRI) Ethics committees and Uganda National Council for Science and Technology.

5. In 2014 and 2018, measures of physical violence include being slapped with a hand, having an ear twisted, hit with an object (incl. caning), doing forced labour, made to stand or kneel, and severely beaten up. In 2014 only, also included being hurt or caused pain, arm twisted, hair pulled, fingers crushed, head knocked, hit with a fist, kicked, burnt, food taken away, choked, tied up, and cut.

6. OR = Odds Ratio, which is the odds of experiencing past year teacher physical violence in one group compared to the odds of experiencing past year teacher physical violence in the reference group, unadjusted for any confounding variables.
